# Obesity and diabetes mellitus are associated with SARS-CoV-2 outcomes without influencing signature genes of extrapulmonary immune compartments at the RNA level

**DOI:** 10.1016/j.heliyon.2024.e24508

**Published:** 2024-01-14

**Authors:** Jöran Lücke, Marius Böttcher, Mikolaj Nawrocki, Nicholas Meins, Josa Schnell, Fabian Heinrich, Franziska Bertram, Morsal Sabihi, Philipp Seeger, Marie Pfaff, Sara Notz, Tom Blankenburg, Tao Zhang, Jan Kempski, Matthias Reeh, Stefan Wolter, Oliver Mann, Marc Lütgehetmann, Thilo Hackert, Jakob R. Izbicki, Anna Duprée, Samuel Huber, Benjamin Ondruschka, Anastasios D. Giannou

**Affiliations:** a Section of Molecular Immunology and Gastroenterology, I. Department of Medicine, University Medical Center Hamburg-Eppendorf, Hamburg, 20246, Germany; b Hamburg Center for Translational Immunology (HCTI), University Medical Center Hamburg-Eppendorf, Hamburg, 20246, Germany; c Department of General, Visceral and Thoracic Surgery, University Medical Center Hamburg-Eppendorf, Hamburg, 20246, Germany; d I. Department of Medicine, University Medical Center Hamburg-Eppendorf, Hamburg, 20246, Germany; e Institute of Legal Medicine, University Medical Center Hamburg-Eppendorf, Butenfeld 34, 22529, Hamburg, Germany; f Institute of Medical Microbiology, Virology, and Hygiene, University Medical Center Hamburg-Eppendorf, Hamburg, Germany; g The Calcium Signaling Group, Department of Biochemistry and Molecular Cell Biology, University Medical Center Hamburg-Eppendorf, Hamburg, 20246, Germany

## Abstract

The severe acute respiratory syndrome coronavirus 2 (SARS-CoV-2) which is responsible for eliciting Coronavirus disease 2019 (COVID-19) still challenges healthcare services worldwide. While many patients only suffer from mild symptoms, patients with some pre-existing medical conditions are at a higher risk for a detrimental course of disease. However, the underlying mechanisms determining disease course are only partially understood. One key factor influencing disease severity is described to be immune-mediated. In this report, we describe a post-mortem analysis of 45 individuals who died from SARS-CoV-2 infection. We could show that although sociodemographic factors and premedical conditions such as obesity and diabetes mellitus reduced survival time in our cohort, they were not associated with changes in the expression of immune-related signature genes at the RNA level in the blood, the gut, or the liver between these different groups. Our data indicate that obesity and diabetes mellitus influence SARS-CoV-2-related mortality, without influencing the extrapulmonary gene expression of immunity-related signature genes at the RNA level.

## 1 Introduction

Since its first occurrence, the global COVID-19 pandemic has cost millions of lives [1]. Despite the successful development of multiple vaccines, the continuous emergence of new variants and scarcely understood long-term health damages pose a continuing threat to global health and the economy alike [2,3]. Aggravated cases of SARS-CoV-2 infection are often associated with a cytokine storm, describing increased concentrations of pro-inflammatory mediators such as interleukin (IL)-1β, IL-6, IL-8, or tumor necrosis factor-alpha (TNFα) [4]. Older individuals, males, and those with comorbidities, such as diabetes mellitus or obesity, have been reported to be at risk of aggravated cases of COVID-19, even after vaccination. The underlying mechanisms creating these differences might be related to latent or chronic inflammation in these conditions.

Furthermore, immunosenescence and inflammaging have been assumed to be responsible for severe COVID-19 outcomes in the elderly [5,6]. Correspondingly, metabolic syndrome also represents a state of chronic inflammation. In obese people, visceral fat contains not only adipocytes but also infiltrating immune cells. These cells have been described to be a major source of cytokines with pro-inflammatory functions. During viral infections, such as SARS-CoV-2 infection, patients suffering from metabolic syndrome have an even higher production of proinflammatory cytokines (such as IL-6 or TNFα) [7] and are consequently prone to a more severe disease outcome [8]. Another facet of metabolic syndrome is diabetes mellitus, which has equally been described to cause chronic inflammation and thereby can potentially promote the occurrence of a fatal cytokine storm in COVID-19-infected patients [9].

Most recently, sex-based differences during SARS-CoV-2 infection have also been stated to be mediated by the differing immune responses between sexes. Explicitly, female individuals have been shown to provide a stronger immune response to different types of infection, including SARS-CoV-2, that is most likely mediated by genetic and hormonal differences [10,11].

Taken together, sociodemographic factors such as sex, age, and pre-existing medical conditions influence the outcome and mortality of SARS-CoV-2 infection. Often enough, they are accompanied by significant changes in the systemic immune response. However, whether these variables also lead to changes in extrapulmonary immune compartments such as the hepatic or intestinal immune compartment, still needs to be determined. This question is of high clinical relevance since multiple studies found associations between intestinal and hepatic SARS-CoV-2 infection and survival. More profound knowledge concerning this topic might also help identify patients at risk for fatal disease courses during future pandemics or might point towards appropriate patient cohorts that profit from treatment with anti-inflammatory drugs.

Here, we investigated a post-mortem cohort of 45 individuals who died from SARS-CoV-2 infection. As countless studies have already investigated the effect of SARS-CoV-2 on lung tissue [12], this study aimed to determine the effect of extrapulmonary SARS-CoV-2 infection in the light of metabolic diseases such as diabetes mellitus and obesity. Thus, we sought to focus on the immune response in other organs mainly responsible for pathological glucose metabolism as present in obesity and diabetes mellitus, namely the liver, responsible for glucose metabolism, and glucose storage and the intestine, responsible for glucose uptake. Of note, due to its high autolytic potential even at comparably low post-mortem intervalls [13], we did not take tissue samples from the pancreas.

By investigating this cohort, we have already confirmed that high expression of hepatic *TNFA* and low expression of intestinal *IL1B* are associated with a reduced survival time [14,15]. Now, using this previously published cohort, we show that obesity and diabetes mellitus, but not sex or age, were associated with a reduced timespan between infection and death. However, after determining the RNA expression of 15 different cytokines and immunological transcriptional factors in the liver, small intestine, and blood, we could not find any significant differences in local cytokine and transcription factor expression levels in patient subgroups divided according to obesity and diabetes mellitus. Only mild differences were observed when samples were divided according to the individual's age and sex. Thus, factors other than only the immune response in the blood, the intestine, or the liver might contribute to the observed survival effects dependent on obesity and diabetes mellitus.

## 2 Methods

### 2.1 Autopsies and acquisition of clinical data

Data acquisition was performed between April 2020 and April 2021. Of all patients included in the study, 45 patients who died from COVID-19 were admitted to the Institute of Legal Medicine. Bodies of the deceased were kept at 4 °C until the scientific autopsy and samples could be taken for this report. Blood was drawn from the femoral vein, while a 1 × 1x1cm tissue sample was taken from the duodenum and the liver from the same location of the organ in every dissection. Subsequently, samples were snap-frozen and stored for further processing. Autopsies were performed following guidelines issued by the German Society for Forensic Medicine. Subsequently, the cause of death was determined as described in more detail elsewhere [16]. Importantly, other causes of death except SARS-CoV-2 infection were excluded from further analytic steps, as were corpses with advanced putrefactive changes. Upon arrival of the samples to the lab, a reverse transcription-quantitative polymerase chain reaction was carried out on nasopharyngeal swab samples at the Institute of Microbiology, Virology, and Hygiene. Next, tissue samples that had been taken from the blood, intestine (duodenum), and liver were snap-frozen in liquid nitrogen. Duodenum was taken as a representative sample of the small intestine as the biopsy location could be determined more precisely between patients due to the duodenum's short length than when using other parts of the small intestine. The clinical data connected to the patients were collected from different sources while the informed consent of legal representatives or relatives was obtained in every case. In detail, demographic data included sex, BMI, age, and the place of death. Specific data concerning the ethnicity of the patients was not collected. Typically, almost all of the investigated patients were not only Caucasian but originated from Hamburg itself. Collected medical data included days of general hospitalization (if any), days of hospitalization in ICU (if any), mechanical ventilation (yes/no), ECMO ventilation (yes/no), gastrointestinal and respiratory symptoms (yes/no), premedical conditions (if any) and laboratory values upon hospital admission (if admitted), and last laboratory values before death. For further analysis, the patient cohort was divided four times in total into two subgroups, each according to different variables. First, in two groups above or below the median of the BMI (median: 26.15, range: 17.8–53.3), second, grouped according to age, (median: 79, range: 4–95), third, in two groups of patients suffering from either diabetes (yes: 19, no: 25) or not and fourth, according to the sex (female: 16, male: 29) of the patients. The study was approved by the Ethics Committee of the Hamburg Chamber of Physicians (reference numbers PV7311 and 2020-10353-BO-ff) and was carried out in accordance with the guidelines of Helsinki.

### 2.2 Quantitative RT-PCR for SARS-CoV-2 detection

In general, the following protocol was carried out as previously reported [17]. In short, the tissue samples were ground together with ceramic beads (Precellys Lysing Kit) and PBS. A part of the lysate was then used for RNA extraction using the MagnaPure96 (Roche, Mannheim, Germany). Primer (5′-ACAGGTACGTTAATAGTTAATAGCmGT-3′, 400 nM end concentration; 5′ TATTGCAGCAGTACGCACAmCA-3′, 400 nM end concentration) and probe (5′-Fam- ACACTAGCC/ZEN/ATCCTTACTGCGCTTCG-Iowa Black FQ-3′, 100 nM end concentration) were added and acquired using Integrated DNA Technologies (IDT, Leuven, Belgium). One-step RT-PCR was carried out by the LightCycler480 system (Roche), using a one-step RNA control kit (Roche) as the master mix and 5 μl of the eluate. The C_T_ value for SARS-CoV-2 RNA was acquired by the second derivative maximum method. Standard *in vitro* transcribed RNA (IVT- RNA) of the E gene of SARS-CoV-2 was used for quantification. The standard was purchased via the European Virus Archive. Of note, the linear range of the assay is between 1 × 10^3^ and 1 × 10^9^ copies/ml. Quantitative β-globin PCR was performed with the respective TaqMan primers (Thermo-Fischer, 401846) and the DNA control kit (Roche). Samples were measured with the LightCycler480 system. Finally, the amount of DNA was normalized using human DNA standard (KR0454), and SARS-CoV-2 RNA levels in tissues were normalized to β-globin DNA to adjust for differences in tissue input.

### 2.3 Quantitative RT-PCR for the detection of cytokines and transcription factors

RNA from tissues was isolated with the Rneasy® Plus Mini Kit (Qiagen, Hilden, Germany) in accordance with the manufacturer's instructions. Samples were adjusted to RNA yield before the high-capacity cDNA synthesis kit (Applied Biosystems, Waltham, US) was used for cDNA synthesis. Finally, real-time PCR was performed by the Kapa Probe Fast qPCR Master Mix (Kapa Biosystems, Wilmington, US) on the StepOne Plus system (Applied Biosystems). All probes were acquired from Applied Biosystems (Table 1). Of note, the relative RNA expression was normalized to *HPRT* and was then calculated by the 2-ΔΔCt method. Due to limited sample availability and retrieved RNA volume, we decided to use a single housekeeping gene only. We chose HPRT as it was identified as the single best housekeeping gene in a study investigating a variety of healthy and tumorous human tissues before [18].

**Table 1:** Probes used for human RNA quantification with RT-qPCR.

Gene	Probe name
*FOXP3*	Hs01058534_m1
*HPRT1*	Hs02800695_m1
*IFNA2*	Hs00265051_s1
*IFNB1*	Hs01077958_s1
*IFNG*	Hs00989291_m1
*IL1B*	Hs00174097_m1
*IL6*	Hs00174131_m1
*IL10*	Hs00961622_m1
*IL17A*	Hs00174383_m1
*IL17F*	Hs00369400_m1
*IL22*	Hs01574154_m1
*IL23*	Hs00900828_g1
*RORC*	Hs01076122_m1
*TBX21*	Hs00203436_m1
*TGFB1*	Hs00998133_m1
*TNF*	Hs01113624_g1

### 2.4 Statistical analysis

The analysis of categorical variables was made by Fisher's exact or Chi-square test. Continuous variables were analysed by the Wilcoxon rank sum test with continuity correction, and the obtained p-values for the cytokine and transcription factor concentrations were adjusted for a false discovery rate of 5 %. The group characteristics were compared with a 2-sample Z-test and p-values adjusted for a false discovery rate of 5 %. Survival estimates were analysed by the Kaplan-Meier method and were subsequently analysed by the log-rank test. *P*-values <0.05 were considered statistically significant. The statistical analysis was performed with STATA/MP, Version 17.0 (StataCorp, Texas, USA) and R, Version 4.2.1 (R Foundation, Vienna, Austria). GraphPad Prism software version 9.1.1 (GraphPad Software, CA, USA) was used for the graphical depiction of the data.

## 3 Results

Different survival rates in BMI^high^ and BMI_low_ groups are not associated with a distinct immune response at the RNA level in blood, intestine, or liver.

**Fig. 1:**
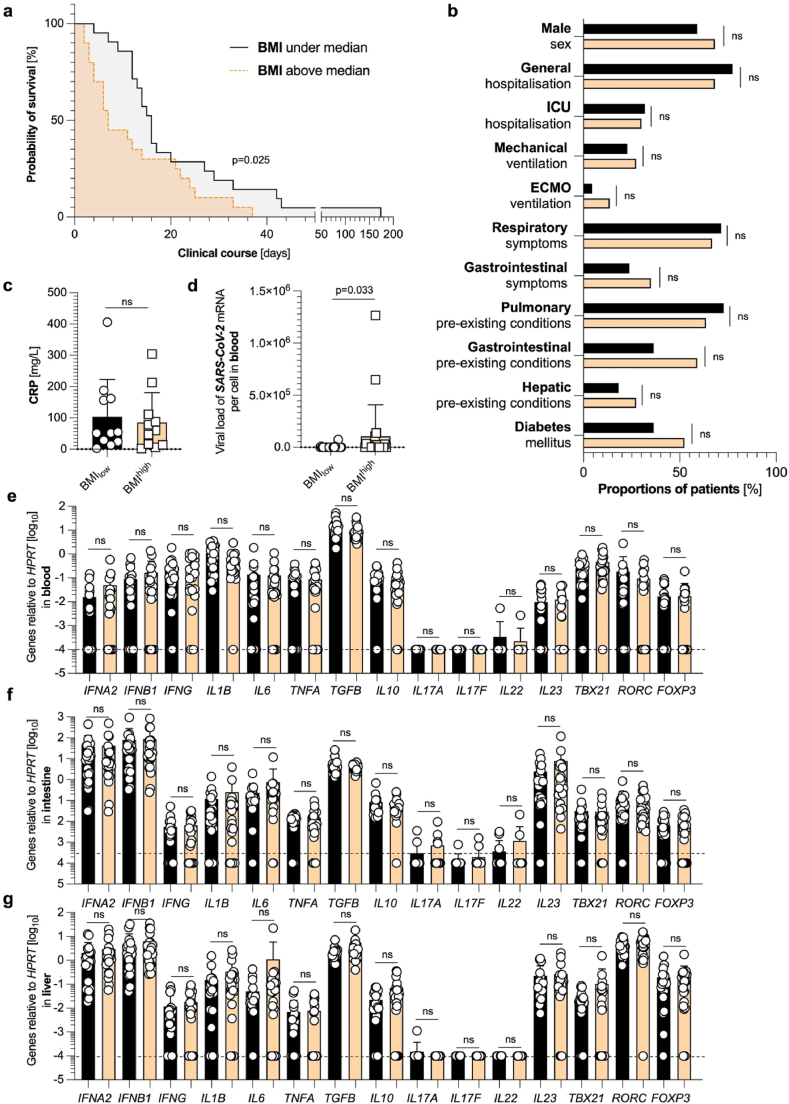
**Different survival in BMI**^**high**^**and BMI**_**low**_**groups is not associated with a distinct immune response in blood, intestine, or liver.** (**a**) Kaplan Meier analysis of survival length in different cohorts divided according to lower (below the median) BMI (n = 22, black), and higher (above the median) BMI (n = 22, yellow). One patient had to be excluded due to missing BMI data. (**b**) Cohort characteristics of patients with lower (below the median) BMI (n = 22, black), and higher (above the median) BMI (n = 22, yellow) are depicted. (**c**) First available CRP levels (in mg/L) upon SARS-CoV-2 diagnosis, determined by screening of patient's laboratory findings, divided according to lower (below the median) BMI (n = 11, black), and higher (above the median) BMI (n = 11, yellow). Statistics by Mann-Whitney *U* test. (**d**) Absolute expression of SARS-CoV-2 mRNA to β-globin DNA in the blood, as measured by RT-PCR, divided according to lower (below the median) BMI (n = 22, black), and higher (above the median) BMI (n = 21, yellow). One blood sample had to be excluded due to undetectable *HPRT* values. (**e**) Relative expression of *IFNA2*, *IFNB1*, *IFNG*, *IL1B*, *IL6*, *TNFA*, *TGFB*, *IL10*, *IL17A*, *IL17F*, *IL22*, *IL23*, *TBX21*, *RORC*, and *FOXP3* mRNA in the blood, as measured by reverse transcriptase polymerase chain reaction (RT-PCR), divided according to lower (below the median) BMI (n = 22, black), and higher (above the median) BMI (n = 21, yellow). One blood sample had to be excluded due to undetectable *HPRT* values. (**f**) Relative expression of *IFNA2*, *IFNB1*, *IFNG*, *IL1B*, *IL6*, *TNFA*, *TGFB*, *IL10*, *IL17A*, *IL17F*, *IL22*, *IL23*, *TBX21*, *RORC*, and *FOXP3* mRNA in the intestine, as measured by reverse transcriptase polymerase chain reaction (RT-PCR), divided according to lower (below the median) BMI (n = 22, black), and higher (above the median) BMI (n = 22, yellow). (**g**) Relative expression of *IFNA2*, *IFNB1*, *IFNG*, *IL1B*, *IL6*, *TNFA*, *TGFB*, *IL10*, *IL17A*, *IL17F*, *IL22*, *IL23*, *TBX21*, *RORC*, and *FOXP3* mRNA in the liver, as measured by reverse transcriptase polymerase chain reaction (RT-PCR), divided according to lower (below the median) BMI (n = 21, black), and higher (above the median) BMI (n = 22, yellow). One liver sample had to be excluded due to undetectable *HPRT* values. The dashed black lines represent the limits of detection. Horizontal lines represent means ± SEM; each symbol indicates one sample from one patient.

First, to investigate the association of body weight with the overall survival time of COVID-19-deceased, we divided our patient cohort according to the median body mass index (BMI). Of note, the median patient age of this cohort was 79 years, while 16 out of 45 examined deceased were female (Table 2). Further clinical characteristics of this cohort were published in a previous publication by the author's group [15]. Indeed, the BMI^high^ group had a significantly decreased time of survival compared to their BMI_low_ counterpart (Fig. 1a). When comparing different characteristics of these two groups, such as sex, pre-medical conditions, hospitalization, admission to ICU, or the requirement of mechanical ventilation, no significant differences could be found (Fig. 1b). In line with these findings, there was not a significant difference in CRP levels in the first drawn blood sample upon SARS-CoV-2 diagnosis (Fig. 1c). However, the post-mortem SARS-CoV-2 viral load in the blood was significantly increased in the BMI^high^ group (Fig. 1d). All other investigated laboratory findings did not display significant differences (data not shown).

**Table 2:** Extended patient characteristics of the investigated cohort.

Patient number	Sex	Age	BMI	PMI (in days)
1	Female	4	26.0	1
2	Female	59	30.9	4
3	Female	70	29.5	6
4	Female	72	28.6	4
5	Female	86	30.6	3
6	Female	88	23.9	3
7	Female	91	22.2	2
8	Female	95	25.7	3
9	Male	47	43.4	3
10	Male	49	25.8	1
11	Male	50	32.2	1
12	Male	64	23.2	6
13	Male	71	23.8	6
14	Male	72	22.3	7
15	Male	75	28.6	1
16	Male	76	29.0	3
17	Male	76	n.d.	2
18	Male	79	24.2	3
19	Male	80	23.9	3
20	Male	84	26.3	4
21	Male	85	26.1	4
22	Male	86	23.7	2
23	Male	88	22.8	4
24	Male	91	23.9	3
25	Male	93	26.2	6
26	Female	56	36.5	2
27	Female	74	25.4	8
28	Female	80	30.0	7
29	Female	84	25.8	4
30	Female	84	27.0	1
31	Female	85	24.9	8
32	Female	94	18.0	3
33	Female	95	20.6	2
34	Male	61	40.2	1
35	Male	62	26.6	2
36	Male	67	33.5	2
37	Male	74	36.1	1
38	Male	75	17.8	8
39	Male	75	33.4	3
40	Male	79	36.2	2
41	Male	80	41.7	3
42	Male	81	34.9	5
43	Male	82	21.6	2
44	Male	88	20.8	2
45	Male	81	53.3	3

We next investigated the cytokine and transcription factor profiles of these two subgroups of individuals. We did not only determine the expression of type-I-interferons (such as *IFNA2* and *IFNB1*), that play a pivotal role in SARS-CoV-2 infection, but also cytokines and transcription factors related to T_H_1 (such as *IFNG*, *TNFA,* and *TBX21*), T_H_17 (such as *IL1B*, *IL6*, *TGFB*, *IL17A*, *IL17F*, *IL22*, *IL23,* and *RORC*), and T_REG_ function and maintenance (such as *IL10* and *FOXP3*). However, no significant difference in RNA expression levels was found between the BMI^high^ group and the BMI_low_ counterpart in the blood (Fig. 1e), the intestine (Fig. 1f), and the liver (Fig. 1g). Taken together, SARS-CoV-2 infection in BMI^high^ and BMI_low_ subgroups of our patient cohort does not present a distinct immune profile at the RNA level based on our 15 pre-selected genes despite a difference in survival time.

Neither clinical differences, nor distinct systemic, intestinal, or hepatic immune profiles at the RNA level can be found in sex-divided groups.

**Fig. 2:**
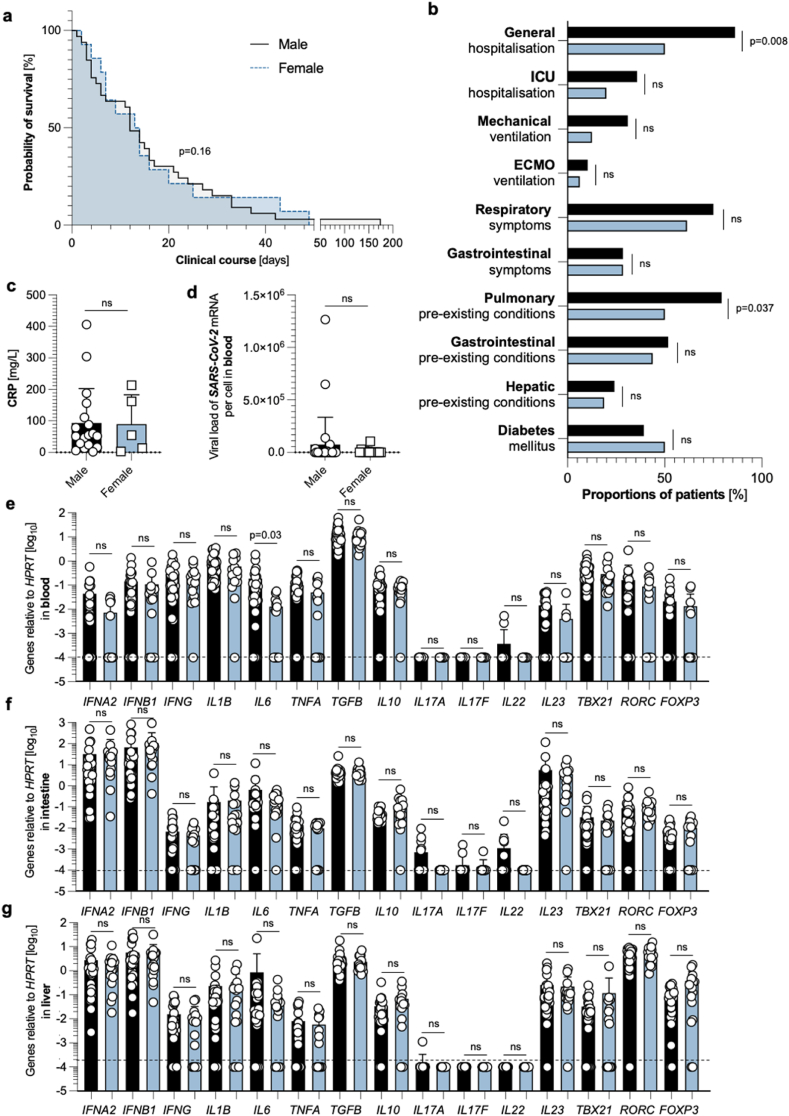
**Neither clinical differences nor distinct systemic, intestinal, or hepatic immune profiles in sex-divided groups.** (**a**) Kaplan Meier analysis of survival length in patient cohorts divided according to male (n = 29, black), and female sex (n = 16, blue). (**b**) Cohort characteristics of male patients (n = 29, black), and female patients (n = 16, blue) are depicted. (**c**) First available CRP levels (in mg/L) upon SARS-CoV-2 diagnosis, determined by screening of patient's laboratory findings, divided according to male (n = 18, black), and female sex (n = 5, blue). Statistics by Mann-Whitney *U* test. (**d**) Absolute expression of SARS-CoV-2 mRNA to β-globin DNA in the blood, as measured by RT-PCR, divided according to male (n = 18, black), and female sex (n = 5, blue). One blood sample had to be excluded due to undetectable *HPRT* values. (**e**) Relative expression of *IFNA2*, *IFNB1*, *IFNG*, *IL1B*, *IL6*, *TNFA*, *TGFB*, *IL10*, *IL17A*, *IL17F*, *IL22*, *IL23*, *TBX21*, *RORC*, and *FOXP3* mRNA in the blood, as measured by reverse transcriptase polymerase chain reaction (RT-PCR), divided according to male (n = 29, black), and female sex (n = 15, blue). One blood sample had to be excluded due to undetectable *HPRT* values. (**f**) Relative expression of *IFNA2*, *IFNB1*, *IFNG*, *IL1B*, *IL6*, *TNFA*, *TGFB*, *IL10*, *IL17A*, *IL17F*, *IL22*, *IL23*, *TBX21*, *RORC*, and *FOXP3* mRNA in the intestine, as measured by reverse transcriptase polymerase chain reaction (RT-PCR), divided according to male (n = 29, black), and female sex (n = 16, blue). (**g**) Relative expression of *IFNA2*, *IFNB1*, *IFNG*, *IL1B*, *IL6*, *TNFA*, *TGFB*, *IL10*, *IL17A*, *IL17F*, *IL22*, *IL23*, *TBX21*, *RORC*, and *FOXP3* mRNA in the liver, as measured by reverse transcriptase polymerase chain reaction (RT-PCR), divided according to male (n = 28, black), and female sex (n = 16, blue). One liver sample had to be excluded due to undetectable *HPRT* values. The dashed black lines represent the limits of detection. Horizontal lines represent means ± SEM; each symbol indicates one sample from one patient.

Next, we wanted to investigate the potential sex-dependent effects of SARS-CoV-2 infection. For this purpose, we divided our cohort according to sex, however, no significant differences were found in the survival time between male and female patients (Fig. 2a). Interestingly, these two cohorts differed in their rate of general hospitalization, as male patients were found to be hospitalized more often (Fig. 2b). Moreover, males also displayed a significant increase in pre-existing pulmonary conditions compared to females but did not differ significantly in further general and clinical characteristics (Fig. 2b). Furthermore, no significant differences were found in CRP levels upon SARS-CoV-2 diagnosis (Fig. 2c), and post-mortem SARS-CoV-2 viral load in the blood could be detected (Fig. 2d). All other investigated laboratory findings also did not display significant differences (data not shown). Finally, except for a reduction of *IL6* levels in the blood of females, cytokine- and transcription factor profiles at the RNA level did not differ between the sexes in the blood (Fig. 2e), the intestine (Fig. 2f), or the liver (Fig. 2g). To sum up, sex neither influences the survival time in our cohort nor does it lead to a significantly different immune profile at the RNA level based on the 15 pre-selected genes in our cohort.

Neither clinical differences nor distinct systemic, intestinal, or hepatic immune profiles at the RNA level can be found in age-divided groups.

**Fig. 3:**
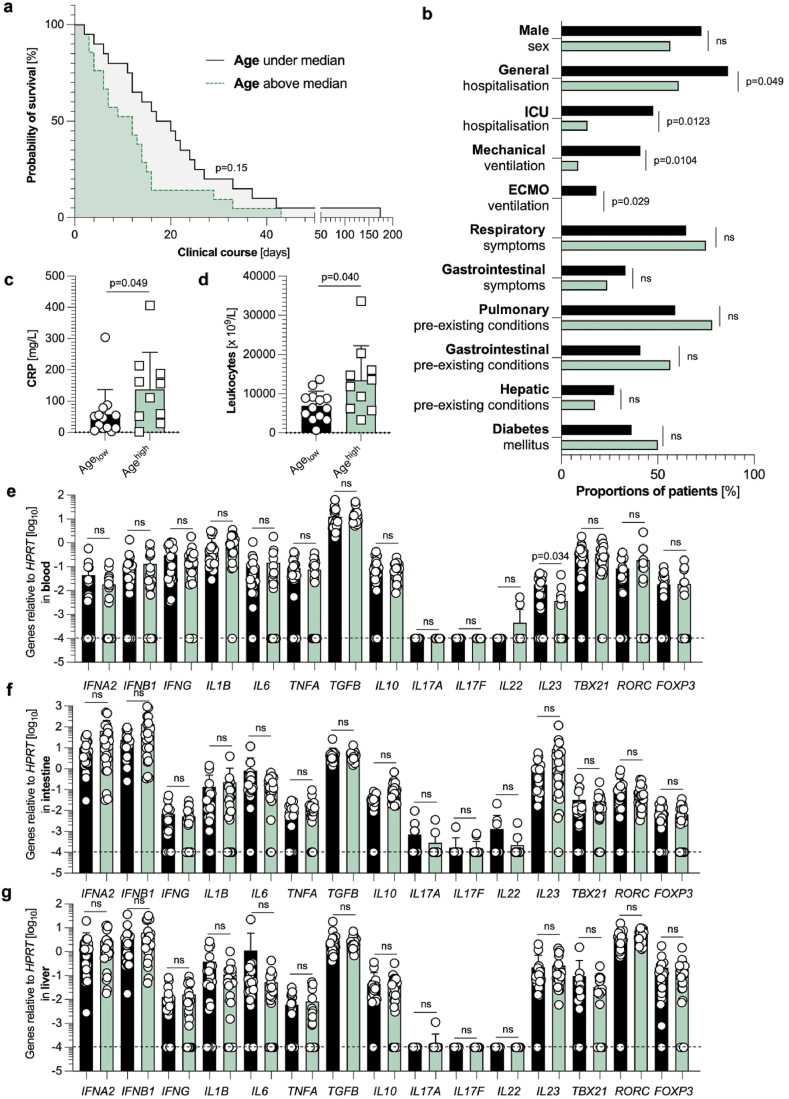
**Neither clinical differences nor distinct systemic, intestinal, or hepatic immune profiles in age-divided groups.** (**a**) Kaplan Meier analysis of survival length in patient cohorts divided according to lower (below the median) age (n = 22, black), and higher (above the median) age (n = 23, green). (**b**) Cohort characteristics of patients with lower (below the median) age (n = 22, black), and higher (above the median) age (n = 23, green) are depicted. (**c**) First available CRP levels (in mg/L) upon SARS-CoV-2 diagnosis, determined by screening of patient's laboratory findings, divided according to lower (below the median) age (n = 13, black), and higher (above the median) age (n = 10, green). Statistics by Mann-Whitney test. (**d**) First available leucocyte count (x 10^9^/L) upon SARS-CoV-2 diagnosis, determined by screening of the patient's laboratory findings, divided according to lower (below the median) age (n = 13, black), and higher (above the median) age (n = 10, green). Statistics by Mann-Whitney *U* test. (**e**) Relative expression of *IFNA2*, *IFNB1*, *IFNG*, *IL1B*, *IL6*, *TNFA*, *TGFB*, *IL10*, *IL17A*, *IL17F*, *IL22*, *IL23*, *TBX21*, *RORC*, and *FOXP3* mRNA in the blood, as measured by reverse transcriptase polymerase chain reaction (RT-PCR), divided according to lower (below the median) age (n = 22, black), and higher (above the median) age (n = 22, green). One blood sample had to be excluded due to undetectable *HPRT* values. (**f**) Relative expression of *IFNA2*, *IFNB1*, *IFNG*, *IL1B*, *IL6*, *TNFA*, *TGFB*, *IL10*, *IL17A*, *IL17F*, *IL22*, *IL23*, *TBX21*, *RORC*, and *FOXP3* mRNA in the intestine, as measured by reverse transcriptase polymerase chain reaction (RT-PCR), divided according to lower (below the median) age (n = 22, black), and higher (above the median) age (n = 23, green). (**g**) Relative expression of *IFNA2*, *IFNB1*, *IFNG*, *IL1B*, *IL6*, *TNFA*, *TGFB*, *IL10*, *IL17A*, *IL17F*, *IL22*, *IL23*, *TBX21*, *RORC*, and *FOXP3* mRNA in the liver, as measured by reverse transcriptase polymerase chain reaction (RT-PCR), divided according to lower (below the median) age (n = 21, black), and higher (above the median) age (n = 23, green). One liver sample had to be excluded due to undetectable *HPRT* values. The dashed black lines represent the limits of detection. Horizontal lines represent means ± SEM; each symbol indicates one sample from one patient.

In the next step, age-dependent influences on the survival time after SARS-CoV-2 infection were investigated. Thus, we divided the present patient cohort according to the median age at the time of death. In our cohort, the Age^high^ group did not have a significantly decreased time of survival compared to the Age_low_ group (Fig. 3a), although several clinical and sociodemographic variables differed significantly between those two groups (Fig. 3b). Likewise, CRP levels upon SARS-CoV-2 diagnosis were significantly increased in the Age^high^ group (Fig. 3c), as were their leucocyte counts (Fig. 3d), suggesting an increased inflammatory response in the elderly patients in our cohort. Once again, all other investigated laboratory findings did not display significant differences (data not shown). However, when comparing the immune profile of our selected cytokines and transcription factors at the RNA level, no clear differences could be detected in the blood (except a significant downregulation of *IL23* expression in the elderly) (Fig. 3e), the intestine (Fig. 3f) or the liver (Fig. 3g). In summary, SARS-CoV-2 infection in the Age^high^ and Age_low_ subgroups of our patient cohort is neither associated with a difference in length of survival time nor can clear differences in the immune profile of our investigated targets at the RNA level be found based on the 15 pre-selected genes.

Different survival times in patients with and without diabetes mellitus are not associated with a distinct immune response at the RNA level in the blood, intestine, or liver.

**Fig. 4:**
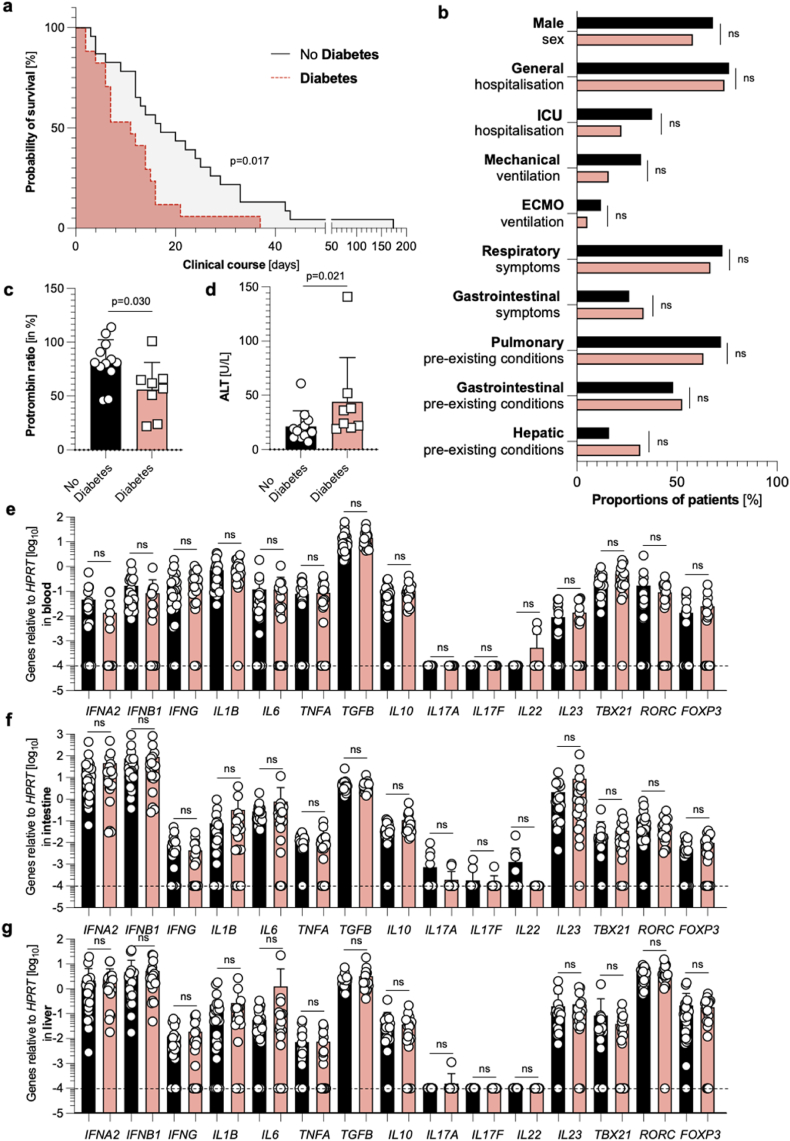
**Different survival in patients with and without diabetes mellitus is not associated with a distinct immune response in the blood, intestine, or liver.** (**a**) Kaplan Meier analysis of survival length in patients without diabetes mellitus (n = 25, black), and with diabetes mellitus (n = 19, red). One patient had to be excluded due to missing information regarding the diabetes mellitus status. (**b**) Cohort characteristics of patients without diabetes mellitus (n = 25, black), and with diabetes mellitus (n = 19, red) are depicted. (**c**) First available prothrombin ratio (in %) upon SARS-CoV-2 diagnosis, determined by screening of patient's laboratory findings, of patients without diabetes mellitus (n = 12, black), and with diabetes mellitus (n = 8, red). Statistics by Mann-Whitney *U* test. (**d**) First available ALT levels (in U/L) upon SARS-CoV-2 diagnosis, determined by screening of patient's laboratory findings, of patients without diabetes mellitus (n = 12, black), and with diabetes mellitus (n = 8, red). Statistics by Mann-Whitney *U* test. (**e**) Relative expression of *IFNA2*, *IFNB1*, *IFNG*, *IL1B*, *IL6*, *TNFA*, *TGFB*, *IL10*, *IL17A*, *IL17F*, *IL22*, *IL23*, *TBX21*, *RORC*, and *FOXP3* mRNA in the blood, as measured by reverse transcriptase polymerase chain reaction (RT-PCR), of patients without diabetes mellitus (n = 25, black), and with diabetes mellitus (n = 18, red). One blood sample had to be excluded due to undetectable *HPRT* values. (**f**) Relative expression of *IFNA2*, *IFNB1*, *IFNG*, *IL1B*, *IL6*, *TNFA*, *TGFB*, *IL10*, *IL17A*, *IL17F*, *IL22*, *IL23*, *TBX21*, *RORC*, and *FOXP3* mRNA in the intestine, as measured by reverse transcriptase polymerase chain reaction (RT-PCR), of patients without diabetes mellitus (n = 25, black), and with diabetes mellitus (n = 19, red). (**g**) Relative expression of *IFNA2*, *IFNB1*, *IFNG*, *IL1B*, *IL6*, *TNFA*, *TGFB*, *IL10*, *IL17A*, *IL17F*, *IL22*, *IL23*, *TBX21*, *RORC*, and *FOXP3* mRNA in the liver, as measured by reverse transcriptase polymerase chain reaction (RT-PCR), of patients without diabetes mellitus (n = 24, black), and with diabetes mellitus (n = 19, red). One liver sample had to be excluded due to undetectable *HPRT* values. The dashed black lines represent the limits of detection. Horizontal lines represent means ± SEM; each symbol indicates one sample from one patient.

Finally, we wanted to investigate the influence of diabetes on survival time. Indeed, patients suffering from this pre-existing medical disease had a significantly reduced survival time (Fig. 4a). Of note, no differences in clinical and sociodemographic characteristics could be detected between patients with and patients without diabetes (Fig. 4b). When investigating the first blood results after SARS-CoV-2 diagnosis, we did not find significantly different values for systemic inflammation (such as CRP or leucocytes; data not shown). However, patients with pre-existing diabetes mellitus had a significantly reduced prothrombin ratio (Fig. 4c), and significantly elevated ALT levels (Fig. 4d), which might point toward increased liver damage in this group. Of note, all other investigated laboratory findings did not display significant differences (data not shown). Nonetheless, the expression of cytokine and transcription factors at the RNA level between the patients with and without diabetes did not differ in the blood (Fig. 4e), the intestine (Fig. 4f), or the liver (Fig. 4g). Taken together, SARS-CoV-2 infection in patients with diabetes mellitus leads to a reduced survival time but is not associated with a distinct immune profile at the RNA level in the blood, the intestine, or the liver in our cohort based on our 15 pre-selected targets.

## 4 Discussion

Despite multiple vaccines, the continuous emergence of new variants, and only partially understood long-term health issues, SARS-CoV-2 infection remains a global threat with the continuous need for further research. While a dysregulated systemic immune system is an essential and well-described feature of severe COVID-19 courses, the implications on the immune response in patients with pre-existing medical conditions and divided by sociodemographic factors are only primitively understood. It is well described that a dysfunctional immune response during SARS-CoV-2 infection may trigger the production of pro-inflammatory mediators, potentially initiating a detrimental cytokine storm [19,20]. In particular, elevated levels of IL-6, IL-7, IL-10, IL-17, IL-23, TNFα, IFN-γ, IFNα or IL-1β have been found in severe COVID-19 cases [[21], [22], [23], [24]] and have already profoundly been analysed in recent Meta-Analysis [25]. However, our study focused on RNA levels of different cytokines and transcriptional factors, that yield different information than investigating protein expression in the serum. Moreover, *TBX21* and *FOXP3,* the main transcriptional factors for T_H_1 cells and T_REG_ cells, respectively, have been found to be overexpressed in patients with poor outcomes in COVID-19 [26,27]. These findings implicate that SARS-CoV-2 affects all branches of the immune system and might explain why agents such as dexamethasone, baricitibin, and tocilitib have been proven to alleviate disease course in many cases [28]. With this work, we could confirm that obesity and diabetes mellitus, but not age nor sex, are associated with a decreased chance of survival in a patient cohort of 45 individuals who died from SARS-CoV-2 infection. Despite being associated with an altered function of the immune system in many organs in the literature, we could not find significant differences in the RNA expression levels of 15 cytokines and transcription factors in the blood, the intestine, or the liver.

Overall, immunosenescence and inflammaging may be responsible for the aggravated COVID-19 outcomes in the elderly. The acquisition of a senescence-associated secretory phenotype (SASP) is one feature of senescent immune cells, which then secrete pro-inflammatory cytokines such as IL-6, TNFα, IL-1β, and IL-8 among others [5,6]. It would be obvious that these senescent immune cells and their released cytokines may also be the reason for a more severe disease course in elderly patients. However, we could not detect an increase of *IL6*, *TNFA*, or *IL1B* RNA expression in the blood of the elderly deceased. On the contrary, in our cohort, patients deceased with an age above the median did not even display a reduced survival time. This might be due to the relatively small cohort size of 45 patients. Moreover, the average age of patients in our cohort is 75 years. However, this average age at death was typical in COVID-19 fatalities in this pandemic period [29]. Nonetheless, this obvious bias towards the elderly deceased and the lack of relevant numbers of deaths below a certain threshold might be the reason why we did not detect a difference in survival length, as the Age_low_ group might still have a much higher age on average than in comparable studies. Even so, the observation of no survival difference in a cohort of old (Age_low_) and very old (Age^high^) still bears relevance as high and very high age is an emerging problem in current medicine.

Taken together, there may be different mechanisms explaining aggravated COVID-19 outcomes in older patients. Therefore, immunosuppressant therapy in these patients might need to be evaluated more carefully. Interestingly, there are studies implicating that steroid use in critically ill elderly COVID-19 patients is associated with increased mortality [30]. Future studies will have to focus on identifying other indicators to predict the benefit of immunosuppressant therapy in elderly patients suffering from COVID-19.

In metabolic syndrome, including the entities of diabetes mellitus and obesity, immune cells settled in the visceral fat have been described to be a source of proinflammatory cytokines that are assumed to predispose patients to a more severe course of COVID-19 by multiple studies [8,[31], [32], [33], [34]]. Likewise, in our cohort, a BMI above the median was also associated with a decreased probability of survival. Moreover, obese patients in the cohort had a higher SARS-CoV-2 viral load in their blood. This observation is in line with current literature, in which delayed viral clearance is positively associated with obesity [35]. However, we did not find a difference in the RNA expression of pro-inflammatory cytokines and transcription factors in different tissues. This surprising finding could be corroborated by another study, in which no weight-dependent difference between several cytokines in the serum of critically ill COVID-19 patients could be detected [36]. However, no difference in 40-day mortality could be detected in these groups as well. Nonetheless, this indicates that other mechanisms causing an aggravated disease course in this patient cohort may play a significant role as well. Possible mechanisms have been stated to lay within a restricted pulmonary function [37] or a higher activity of the aldosterone system in obese patients. These findings are supported by the fact that there seems to be no significant difference in survival between obese and normal‐weight groups treated with dexamethasone [38]. However, further studies are warranted to support this hypothesis.

Most recently, sex-based differences in COVID-19 have also been related to differences in the immune response, reporting overall better outcomes in females. This observation could be explained by genetic and hormonal differences, that have been described to lead to a more effective type-I-interferon-response [10,39,40]. Surprisingly, we could neither confirm a significant difference in survival rates between male and female individuals while other results do so [41]. One possible explanation might be the small cohort size compared to other studies. However, we could detect a significantly decreased expression of *IL6* in the blood of COVID-19-infected females, an observation that was also made by others [42]. Otherwise, there was no significant difference in the other 14 pre-selected cytokines and transcription factors in the blood, the intestine, or the liver.

Although the immune system seems to be a major factor determining the outcome after SARS-CoV-2 infection, we could not find a significant difference in cytokine and transcription factor expression at the RNA level in the blood, the intestine, and the liver in patient subgroups divided according to obesity, diabetes mellitus, age, and sex with two exceptions.

However, our study possesses several limitations. First and foremost, we did not measure protein cytokine levels via ELISA but assessed the RNA expression in different compartments. In line with this, RNA and their transcribed products often undergo further posttranscriptional and posttranslational modifications, as it is for example the case for CRP [43]. Thus, the conclusions of this manuscript cannot be extended to protein transcripts of the described inflammatory mediators. Furthermore, we also did not measure antibodies in the serum directed against SARS-CoV-2, so that a serological response and its dependence on obesity and diabetes cannot be determined based on the presented data. Second, our cohort consists only of fatal SARS-CoV-2 infections, and thus, individuals had the most severe course of this disease, which distinguishes our cohort from many others that were investigated clinically. Moreover, post-mortem changes in RNA expression levels can also not be excluded, as inflammatory serum makers are indeed influenced by the post-mortem interval [44,45]. Indeed, Ferreira et al. demonstrated that the RNA profile of tissues significantly changes after death [46] while other studies corroborated the notion of this study by showing that sTNFR1 and IL-6 serum levels increased with longer post-mortem intervals [44,47]. However, we did not detect any correlation between the PMI and any investigated gene at RNA level in all three tissues investigated (data not shown), rendering a major effect of the PMI on our presented results improbable.

Third, we pre-selected 15 common immune-system-related genes, which renders our study design biased by potential default. Further unbiased approaches such as bulk sequencing or OMICS technologies are superior for clarifying the role of sociodemographic factors on the immune system during SARS-CoV-2 infection, however, these methods exceed the scope of our study.

Fourth, the study employed a comparably small cohort of just 45 patients, limiting comprehensive comparisons of sex, age, and BMI and decreasing the ability to draw meaningful conclusions. However, using autopsy tissues, a sample size of 45 is an acceptable case count as other existing autopsy studies from 2020 to 2021 that sampled SARS-CoV-2 tissues worked with equally sized cohorts, too [48].

Moreover, we decided to not investigate the immune cell composition and RNA expression of local cytokines and transcription factors of lung tissue in our cohort, as many studies have performed this analysis before. Nonetheless, this limits the scope of our study significantly. First, differences in lung tissue might reveal potential reasons for the observed differences in survival length that remain hidden otherwise. Second, no associations can be drawn in this study between the relation of intra- and extrapulmonary cytokine expression profiles, which would have added additional information regarding the spatial distribution of inflammatory patterns during SARS-CoV-2 infection.

Taken together, our data present that obesity and diabetes mellitus lead to a reduced survival time during COVID-19. Moreover, no significant changes in the expression pattern of 15 inflammation markers at the RNA level in the blood, the liver, and the intestine could be detected in these groups, implicating that the immune response in other compartments (such as the lung, for example) or non-immune related factors may play a key role in mediating these observations. Nonetheless, precise molecular mechanisms explaining these survival differences in the different groups are still lacking. Therefore, further investigation is needed to provide individualized treatment in these groups.

## Data availability statement

Data will be made available on request.

## CRediT authorship contribution statement

**Jöran Lücke:** Writing – review & editing, Writing – original draft, Visualization, Supervision, Project administration, Data curation, Conceptualization. **Marius Böttcher:** Writing – review & editing, Writing – original draft, Data curation. **Mikolaj Nawrocki:** Software, Investigation, Formal analysis. **Nicholas Meins:** Data curation. **Josa Schnell:** Data curation. **Fabian Heinrich:** Data curation. **Franziska Bertram:** Investigation, Data curation. **Morsal Sabihi:** Writing – review & editing, Data curation. **Philipp Seeger:** Writing – review & editing, Data curation. **Marie Pfaff:** Writing – review & editing, Data curation. **Sara Notz:** Writing – review & editing, Data curation. **Tom Blankenburg:** Data curation. **Tao Zhang:** Data curation. **Jan Kempski:** Data curation. **Matthias Reeh:** Writing – review & editing, Investigation. **Stefan Wolter:** Investigation. **Oliver Mann:** Investigation. **Marc Lütgehetmann:** Methodology, Investigation. **Thilo Hackert:** Investigation. **Jakob R. Izbicki:** Investigation. **Anna Duprée:** Investigation. **Samuel Huber:** Writing – original draft, Investigation, Funding acquisition. **Benjamin Ondruschka:** Writing – review & editing, Writing – original draft, Investigation, Funding acquisition. **Anastasios D. Giannou:** Writing – review & editing, Writing – original draft, Investigation, Funding acquisition, Data curation.

## Declaration of competing interest

The authors declare the following financial interests/personal relationships which may be considered as potential competing interests: Samuel Huber reports financial support was provided by 10.13039/501100001659Deutsche Forschungsgemeinschaft. Samuel Huber reports financial support was provided by Ernst 10.13039/501100004038Jung-Stiftung Hamburg. Samuel Huber reports financial support was provided by Stiftung Experimentelle Biomedizin. Fabian Heinrich reports financial support was provided by NATON project within the Netzwerk Universitätsmedizin. Benjamin Ondruschka reports financial support was provided by NATON project within the Netzwerk Universitätsmedizin. Anastasios Giannou reports financial support was provided by Else Kröner Memorial Stipendium. Anastasios Giannou reports financial support was provided by ung Foundation for Science and Research (Ernst Jung Career Development Award 2022). If there are other authors, they declare that they have no known competing financial interests or personal relationships that could have appeared to influence the work reported in this paper.

## References

[1] Collaborators C.-E.M. (2022). Estimating excess mortality due to the COVID-19 pandemic: a systematic analysis of COVID-19-related mortality, 2020-21. Lancet.

[2] Control E.C.f.D.P.a. (2023). https://www.ecdc.europa.eu/en/covid-19/variants-concern.

[3] Davis H.E., McCorkell L., Vogel J.M., Topol E.J. (2023). Long COVID: major findings, mechanisms and recommendations. Nat. Rev. Microbiol..

[4] Tang Y. (2021). Aberrant cytokine expression in COVID-19 patients: associations between cytokines and disease severity. Cytokine.

[5] Witkowski J.M., Fulop T., Bryl E. (2022). Immunosenescence and COVID-19. Mech. Ageing Dev..

[6] Nacarelli T. (2019). NAD(+) metabolism governs the proinflammatory senescence-associated secretome. Nat. Cell Biol..

[7] Steenblock C. (2021). COVID-19 and metabolic disease: mechanisms and clinical management. Lancet Diabetes Endocrinol..

[8] Seidu S. (2021). The impact of obesity on severe disease and mortality in people with SARS-CoV-2: a systematic review and meta-analysis. Endocrinol Diabetes Metab.

[9] Mehta P. (2020). COVID-19: consider cytokine storm syndromes and immunosuppression. Lancet.

[10] Arnold C.G., Libby A., Vest A., Hopkinson A., Monte A.A. (2022). Immune mechanisms associated with sex-based differences in severe COVID-19 clinical outcomes. Biol. Sex Differ..

[11] Fairweather D., Rose N.R. (2004). Women and autoimmune diseases. Emerg. Infect. Dis..

[12] Milross L. (2022). Post-mortem lung tissue: the fossil record of the pathophysiology and immunopathology of severe COVID-19. Lancet Respir. Med..

[13] Shimizu M., Hayashi T., Saitoh Y., Ohta K., Itoh H. (1990). Postmortem autolysis in the pancreas: multivariate statistical study. The influence of clinicopathological conditions. Pancreas.

[14] Lücke J. (2023). TNFα aggravates detrimental effects of SARS-CoV-2 infection in the liver. Front. Immunol..

[15] Lucke J. (2023). Intestinal IL-1beta plays a role in Protecting against SARS-CoV-2 infection. J. Immunol..

[16] Edler C. (2020). Dying with SARS-CoV-2 infection-an autopsy study of the first consecutive 80 cases in Hamburg, Germany. Int. J. Leg. Med..

[17] Puelles V.G. (2020). Multiorgan and renal Tropism of SARS-CoV-2. N. Engl. J. Med..

[18] de Kok J.B. (2005). Normalization of gene expression measurements in tumor tissues: comparison of 13 endogenous control genes. Lab. Invest..

[19] Hojyo S. (2020). How COVID-19 induces cytokine storm with high mortality. Inflamm. Regen..

[20] Tay M.Z., Poh C.M., Renia L., MacAry P.A., Ng L.F.P. (2020). The trinity of COVID-19: immunity, inflammation and intervention. Nat. Rev. Immunol..

[21] Sharif-Askari F.S. (2022). Interleukin-17, a salivary biomarker for COVID-19 severity. PLoS One.

[22] Laurent P. (2022). Sensing of SARS-CoV-2 by pDCs and their subsequent production of IFN-I contribute to macrophage-induced cytokine storm during COVID-19. Sci Immunol.

[23] Mahallawi W.H., Khabour O.F., Zhang Q., Makhdoum H.M., Suliman B.A. (2018). MERS-CoV infection in humans is associated with a pro-inflammatory Th1 and Th17 cytokine profile. Cytokine.

[24] Galbraith M.D. (2022). Specialized interferon action in COVID-19. Proc. Natl. Acad. Sci. U.S.A..

[25] Hu H. (2022). Increased Circulating cytokines have a role in COVID-19 severity and death with a more Pronounced effect in males: a systematic review and meta-analysis. Front. Pharmacol..

[26] Abdelhafiz A.S. (2021). Upregulation of FOXP3 is associated with severity of hypoxia and poor outcomes in COVID-19 patients. Virology.

[27] Kalfaoglu B., Almeida-Santos J., Tye C.A., Satou Y., Ono M. (2020). T-cell hyperactivation and paralysis in severe COVID-19 infection revealed by single-cell analysis. Front. Immunol..

[28] Lamontagne F. (2020). A living WHO guideline on drugs for covid-19. BMJ.

[29] Lohner L. (2021). [SARS-CoV-2-associated deaths in adult persons up to 50 years of age]. Rechtsmedizin.

[30] Jung C. (2021). Steroid use in elderly critically ill COVID-19 patients. Eur. Respir. J..

[31] Chen G. (2020). Clinical and immunological features of severe and moderate coronavirus disease 2019. J. Clin. Invest..

[32] Singh M. (2022). Type 2 diabetes contributes to altered adaptive immune responses and vascular inflammation in patients with SARS-CoV-2 infection. Front. Immunol..

[33] Hulme K.D., Noye E.C., Short K.R., Labzin L.I. (2021). Dysregulated inflammation during obesity: driving disease severity in influenza Virus and SARS-CoV-2 infections. Front. Immunol..

[34] Hornung F., Rogal J., Loskill P., Loffler B., Deinhardt-Emmer S. (2021). The inflammatory profile of obesity and the role on pulmonary bacterial and viral infections. Int. J. Mol. Sci..

[35] Zhang X. (2021). Delayed SARS-CoV-2 clearance in patients with obesity. Infect. Drug Resist..

[36] Kooistra E.J. (2021). A higher BMI is not associated with a different immune response and disease course in critically ill COVID-19 patients. Int. J. Obes..

[37] Dietz W., Santos-Burgoa C. (2020). Obesity and its implications for COVID-19 mortality. Obesity.

[38] Abouir K. (2022). Dexamethasone exposure in normal-weight and obese hospitalized COVID-19 patients: an observational exploratory trial. Clin Transl Sci.

[39] Morris J.A., Harrison L.M. (2009). Hypothesis: Increased male mortality caused by infection is due to a decrease in heterozygous loci as a result of a single X chromosome. Med. Hypotheses.

[40] Saitoh S.I. (2017). TLR7 mediated viral recognition results in focal type I interferon secretion by dendritic cells. Nat. Commun..

[41] Stanelle-Bertram S. (2023). CYP19A1 mediates severe SARS-CoV-2 disease outcome in males. Cell Rep Med.

[42] Lau E.S. (2021). Sex differences in inflammatory markers in patients hospitalized with COVID-19 infection: insights from the MGH COVID-19 patient registry. PLoS One.

[43] Kim Y. (2015). Posttranscriptional regulation of the inflammatory marker C-reactive protein by the RNA-binding protein HuR and MicroRNA 637. Mol. Cell Biol..

[44] Ondruschka B. (2019). Post-mortem in situ stability of serum markers of cerebral damage and acute phase response. Int. J. Leg. Med..

[45] Ondruschka B., Schuch S., Pohlers D., Franke H., Dressler J. (2018). Acute phase response after fatal traumatic brain injury. Int. J. Leg. Med..

[46] Ferreira P.G. (2018). The effects of death and post-mortem cold ischemia on human tissue transcriptomes. Nat. Commun..

[47] Tsokos M., Reichelt U., Jung R., Nierhaus A., Puschel K. (2001). Interleukin-6 and C-reactive protein serum levels in sepsis-related fatalities during the early postmortem period. Forensic Sci. Int..

[48] Autsch A. (2023). SARS-CoV-2-associated fatalities within the first year of the COVID-19 pandemic: an autopsy study. Rechtsmedizin.

